# Protocol for data collection on the language of poor students in Malaysia^☆^

**DOI:** 10.1016/j.mex.2025.103616

**Published:** 2025-09-11

**Authors:** Wan Athirah Adilah Wan Halim, Muhamad Fadzllah Zaini, Mazura Mastura Muhammad, Mohd Haniff Md.Tahir, Nur Farahkhanna Mohd Rusli, Nurshafawati Ahmad Sani, Habibah Ismail, Suriati Zakaria, Md. Zahril Nizam Md. Yusoff, Norliza Jamaluddin, Dahlia Janan, Darwalis Sazan

**Affiliations:** a Faculty of Languages and Communication, Universiti Pendidikan Sultan Idris, 35900 Tg. Malim, Perak, Malaysia; b Faculty of Languages and Communication, Universiti Sultan Zainal Abidin, 21300 Kuala Nerus, Terengganu, Malaysia; c Faculty of Major Language Studies, Universiti Sains Islam Malaysia, Bandar Baru Nilai, 71800 Nilai, Negeri Sembilan, Malaysia; d SMK Jengka Pusat, 26400 Bandar Jengka, Pahang, Malaysia; e School of Distance Education, Universiti Sains Malaysia, 11800 USM Penang, Malaysia

**Keywords:** Releasing permission, Data collection, Poor students, Methodology, Protocol

## Abstract

Poverty is defined as the lack of financial resources to meet basic needs, including insufficient goods, poor physical health, inadequate food and clothing, the absence of proper housing, and the lack of employment that provides a stable income. This deprivation affects the educational development of students from impoverished backgrounds. To identify and analyze data on extremely poor students, a protocol was developed. This protocol is named the Protocol for Data Collection on the Language of Poor Students in Malaysia. It is divided into three main phases and nine steps to facilitate the data collection process. Collecting data from this vulnerable group requires adherence to specific ethical guidelines, which encompass both educational and human ethics. Compliance with these ethical standards ensures a clearer understanding of the data collected. The data gathered is multi-sourced, involving language (oral and written), economics (income, etc.), social factors (gender, etc.), and geography (location). All these elements contribute to achieving SDG 4 (Quality Education) and support SDG 1 (No Poverty).

Overall, this methodology:

A protocol was designed to collect language data from extremely poor students in Malaysia.

The data collection process adheres to specific ethical guidelines, including educational and human ethics, to ensure the respectful and responsible gathering of sensitive data from vulnerable groups.

The collected data includes various sources such as language (oral and written), socioeconomic factors (income), social factors (gender), and geographical location, allowing for a comprehensive analysis of the factors influencing extreme poverty in educational contexts.

## Graphical abstract

**Figure:**
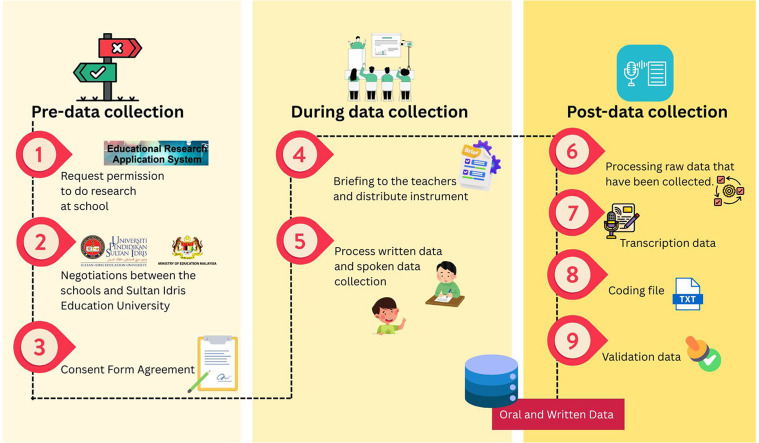

Specifications table

**Table:** 

**Subject area**	Computer Science
**More specific subject area**	*Corpus Linguistics*
**Name of your method**	*Protocol for Data Collection on the Language of Poor Students in Malaysia*
**Name and reference of original method**	*None*

## Background

Every nation has laws and ethical standards that must be followed. When developing a research study, the primary attention shifts to data collection. Data is a crucial output for demonstrating capability trends in all studies. To preserve safety, preparedness, and sensitive topics, several ethics and legality must be followed within the discipline of teaching. Malaysian education research must adhere to the Educational Research System, overseen by the Ministry of Education Malaysia's Planning and Educational Policy Research Division (KPM). Research involving Indigenous students must follow Indigenous Research Ethics guidelines from the Research Division, Department of Indigenous Development (JAKOA). Universities and institutes must follow Human Research Ethics.

The main objective of this study is to establish and describe a comprehensive protocol for data collection related to the language of poor students in Malaysia, with a focus on ensuring ethical and legal compliance during the process. The scope of this study is primarily concerned with outlining the procedures involved in gathering data on the linguistic abilities of students from low socio-economic backgrounds in Malaysian schools. It aims to provide clear guidelines for researchers to follow while conducting studies involving vulnerable groups, ensuring that their work adheres to the ethical standards set by the Ministry of Education Malaysia and other relevant bodies.

Researchers may apply via the Educational Research System's eRAS2.0 (https://eras.moe.gov.my/) platform. The method asks researchers to prepare documentation and complete an online application form. The approval time is two (2) weeks after submission. If there are changes, the system will resend for revisions and corrections. Researchers who achieve study compliance standards must adhere to conditional permission guidelines. Some of the clauses for conditional permission are:

### Conditional completion

*"THIS APPROVAL IS CONTINGENT UPON THE DELIBERATION OF THE DIRECTOR OF THE VOCATIONAL EDUCATION AND TRAINING DIVISION, THE JPN DIRECTOR, AND THE CONSENT OF THE RELEVANT SCHOOL OR VOCATIONAL COLLEGE ADMINISTRATOR."*

This research has been approved at the Ministry of Education Malaysia, State Education Department, District Education Office, and School levels thanks to this procedure. This hierarchy plays an essential role in the ethical approval procedure for conducting educational research in schools. As a result, the research titled "Development of the Relative Poverty Index for Hardcore Poor Based on the Integration of Vocabulary Corpus and Socioeconomics." The purpose of this research is to dive into the background of severe poverty at the basic education level.

Socioeconomic studies and vocabulary are under-represented at the global level. The use of these word markers in poverty measurement remains limited. Mental space has a significant influence on low-income individuals' lifestyles and schooling. When children or other family members have a limited vocabulary, communication and learning at school suffer. According to the research [1], various variables contribute to poor reading performance in children from low socioeconomic backgrounds. Among the factors involved are (1) the interaction of genetics and environment in reading ability; (2) phonological awareness, oral language ability, and vocabulary knowledge: the importance of early home learning environment (HLE) (before school age); (3) phonological awareness (phonological awareness refers to the understanding that spoken language is made up of distinct sound streams); and (4) vocabulary and oral language. Although the link between spoken language (including vocabulary) and reading is well understood, particularly the association between vocabulary and comprehension [3,8,9], the processes at work are still unclear. Oral language influences early reading abilities [4,7]. Farkas and Beron [2] discovered substantial variations in receptive vocabulary across children from various socio-economic categories at age three, which grew by age five and remained steady until age 13. In Malaysia, studies have primarily examined language proficiency, essay writing, and communication levels without considering socio-economic factors ([6]; Sylvester Lau Yueh Wei et al., 2020; [5,11]).

As a result, this methodology will help future researchers perform studies and meet compliance criteria throughout the data gathering phase in the education sector. This differs from published written data, such as books and printed/digital documents, which need copyright clearance [10]. This protocol covers human data, both written and spoken, in its raw form, as well as the gathering and learning procedures for data analysis.

## Method details

The design of this study uses a mixed-methods approach, which includes both qualitative and quantitative methods. This design phase is divided into two, namely the Data Collection and Data Analysis Phases. This paper will focus on the Data Collection Phase that uses a quantitative design.

Through the collaboration with the Department of Statistics Malaysia (DOSM), the study leverages key insights from the 2020 Report on Household Income Estimates and Poverty Incidence in Malaysia, particularly in relation to states with significant poverty concentrations. Given that the research grant was approved for the duration of two years (2022–2024), the data from DOSM for the year 2022 was chosen as the basis for this study.

Generally, poverty can be measured using various methods, including absolute poverty measurement and relative poverty measurement. A household is considered to be in absolute poverty if its income is lower than the Poverty Line Income (PLI) and is insufficient to meet basic needs such as food, clothing, and housing. Extreme poverty, on the other hand, is when a household's income is lower than the food poverty line. Relative poverty, on the other hand, is a condition where a household's income is below half of the median household income. Each country has its own benchmarks for measuring poverty, but the method often used by most upper-middle-income countries, including Malaysia, is the measurement of absolute poverty. This is the main indicator in monitoring poverty eradication.

This study selects five zones that represent critical poverty areas, namely; Northern Zone = Kedah, Malaysia; Central Zone = Perak, Malaysia; Southern Zone = Johor, Malaysia; Eastern Zone = Kelantan, Malaysia; BorneoZone = Sabah, Malaysia and Sarawak, Malaysia. The selection of the five zones is based on DOSM Report.

### Implementation protocol for data collection on the language of poor students in Malaysia

Table 1 the study follows a structured protocol for data collection on the language of poor students in Malaysia. In the pre-data collection phase, the research team seeks permission from the Ministry of Education Malaysia and school administrators, followed by distributing consent forms to parents with the help of teachers. During data collection, teachers are briefed, and students complete written and spoken tasks under the guidance of researchers. Post-data collection involves processing and transcribing the raw data, coding the files for analysis, and validating the data with the help of language experts to ensure accuracy and reliability.

**Table 1:** Study Protocol.

Phase	Step	Methods	Human Involved
***Pre-data collection***	1. Request permission to do research at school	Prepare research proposals, required documents and submit them to the Malaysia Education Ministry and State Education Department.	Officer at the Ministry of Education Malaysia and Department of State Education.
	2. Negotiations between the schools and Sultan Idris Education University	Give notification letter to do research at school involved. *Official Letter Submission* The research team from UPSI sends a formal letter to the school administration to request collaboration for the proposed study. *Initial Briefing Session* A briefing session (online or face-to-face) is conducted to explain the research objectives, school involvement, and proposed timeline. *Discussion and Coordination* Both parties engage in discussions to coordinate suitable dates, times, selected classes, and any specific requirements aligned with the school’s schedule. *Agreement and Documentation* A Letter of Acceptance or Collaboration Agreement is signed by both parties to formalize the agreement. *Follow-up Communication* A designated liaison officer from UPSI maintains consistent communication with the school to ensure smooth implementation of the research activities.	School and University administrators
	3. Consent Form Agreement	Distribute consent form to the parents with the help of the teachers and they have to return the consent form	Teachers, Parents, Students
		*Preparation of Consent Forms* The research team prepares detailed and age-appropriate consent forms for parents/guardians, clearly explaining the study’s purpose, procedures, and ethical considerations. *Distribution via Teachers* Consent forms are distributed to parents through the students, with assistance from the class or subject teachers. *Parental Review and Signature* Parents/guardians are given a few days to review, sign, and return the consent forms to the school. *Collection Before Data Gathering* All signed consent forms are collected by the teachers and handed over to the research team before any data collection begins. *Ethical Filing and Verification* The signed forms are filed securely for record-keeping and ethical compliance, ensuring that only students with returned consent are involved in the study.	
***During data collection***	4. Briefing to the teachers and distribute instrument	Researcher give briefing to the teachers and distribute the instrument for spoken and written data collection	Researcher, Teachers, Students
	5. Process written data and spoken data collection	Students will be given a task according to the appointed time. (Refer instrument details)	Teachers, Students.
		[Written Data] About 1 hour and 30 min, students do the task that has been given. [Spoken Data] About 40 min, teacher and students do the task that has been given. The *'Voice Memo'* application will be utilised to record all spoken data as students completed the assigned task.	
		After finishing, all the tasks will be collected.	
***Post-data collection***	6. Processing raw data that have been collected.	All the data will be tagged one by one :All spoken data will be utilised for the data collection.	Researchers
	7. Transcription data	All the data will be transcript and saved in a folder with txt. format. During the data cleaning process, spelling and punctuation errors (Written Data) will be corrected.	Researchers
	8. Coding file	Coding file with txt. format has been prepared to insert in the corpus software (LancsBoxX by Lancaster University). Oral and Written Data are separated into files for each zone.	Researchers
	9. Validation data	Appoint a language expert to verify the data	Researchers, data validator

Table 2 outlines the key instrument requirements for data collection. The first requirement is Theme Selection, where a suitable topic is chosen from the textbook to ensure it is relevant to students of all age groups. The theme "School Holiday" is selected as it is age-appropriate and encompasses social, cultural, and economic issues, making it engaging for students from diverse backgrounds. The second requirement is Data Selection, which involves collecting written and spoken data from students. This data will be transcribed into txt format for analysis using *LancsboxX* software, allowing for in-depth linguistic analysis. The third requirement, Socioeconomic Information of Students and Families, gathers data on students' socioeconomic backgrounds, including salary level, gender, parents' occupations, and location. This information helps to provide context for understanding how socioeconomic factors might influence students' language use and educational outcomes.

**Table 2:** Instruments requirements for collection data.

Instrument Requirements	Steps	Description
Theme selection	-Sorting topics from the textbook, selecting the most suitable theme for all age groups.	-The theme "School Holiday" is chosen because it is age-appropriate and covers social, cultural, and economic issues relevant to students of various age groups.
Data selection	-Collecting written and spoken data from students, which will be analyzed using LancsboxX software.	-The data will be in the form of essays and spoken responses. Transcriptions of spoken data will be converted into txt format for further analysis.
Socioeconomic Information of Students and Families	-Collect data on the student's socioeconomic background, including salary level, gender, parents' occupations, and location.	-This data provides context for understanding the students' social environment and its potential impact on their language use and educational experience. It helps to correlate socioeconomic factors with educational outcomes.

Table 3 outlines the methods used for collecting both oral and written data. The first method, Classroom Observation (Oral Language), involves observing interactions between teachers and students for 40 min. The second method, Student Examination Paper (Written Language), requires students to answer open-ended essay questions provided by the teacher, which will be written in Malay. This method aims to assess students' vocabulary and language skills.

**Table 3:** Methods used according to the collection of oral and written data.

Method	Explanation
Classroom Observation (Oral Language)	Classroom observation will be conducted with teachers and students for 40 min. 1. Topic Preparation The teacher randomly selects a topic for the lesson to be observed. 2. Recording Equipment Setup The observer ensures that audio recording equipment is ready to capture the interactions between the teacher and students during the session. 3. Coordination with Teacher Discuss with the teacher the time and activities to be observed, as well as the specific aspects to be noted. 4. Informing Students Inform the students that an observation will be conducted, ensuring they are aware but not pressured. 5. Conducting the Observation The observer observes the teacher-student interactions and student engagement, using the audio recording equipment for the 40-minute session. 6. Recording Results Record the language used by the teacher and students, as well as the level of student participation, based on the audio recordings.
Student Examination Paper (Written Language)	This method involves students answering open-ended essay questions provided by the teacher. The aim is to assess vocabulary usage among students. Both the questions and answers will be written in Malay. 1. Theme Selection The teacher selects the theme "School Holiday" for the open-ended essay questions, ensuring it is relevant to students and can be easily adapted to various grade levels. 2. Question Preparation The teacher prepares open-ended essay questions related to the theme "School Holiday," with varying complexity depending on the grade level (e.g., simpler questions for lower grades and more complex ones for higher grades). 3. Instructions to Students The teacher provides clear instructions, emphasizing that the essay must be written in Malay, and explains that the difficulty of the question will match their grade level. 4. Distributing the Questions The teacher distributes the essay questions to the students, ensuring each student receives the correct set of questions for their grade level. 5. Student Response Time Students are given a set amount of time to complete their essays, with the expectation that responses will reflect their grade level in terms of vocabulary and complexity. 6. Collection of Responses Once the essays are completed, the teacher collects the written responses for analysis, ensuring both the questions and answers are in Malay and reflect the appropriate complexity for each grade level.

Table 4 the study uses purposive sampling across five zones, covering primary, secondary, and pre-university schools with 270 poverty-affected students.

**Table 4:** Overall study sample used.

Study Sample	Explanation
Purposive sampling technique	This technique focuses on 5 zones, each representing 3 types of schools that have students from the poverty group. Each school consists of 5 classes, with 3 students/pupils per class representing the relevant status. The study includes three school levels: Primary, Secondary, and Pre-University, with a total of 270 students/pupils.

## Method validation

The protocol employed in this research was meticulously created during the Data Collection Protocol Development Workshop for Underprivileged Students, which brought together specialists in corpus linguistics, human ethics, and educational ethics. The development procedure included the primary researcher as well as researchers with experience in their respective disciplines. The data gathering tool was rigorously evaluated by two panels of specialists with over 20 years of expertise in corpus linguistics and language instruction. These panels evaluated the instrument's appropriateness, reliability, and validity using their practical expertise, assuring that the procedures used in this research are valid, relevant, and capable of producing high-quality data. The instrument's validity is further reinforced by a careful application of ethical principles, such as study participant protection, which ensures that the data gathering method corresponds to acknowledged ethical norms in social and educational research. As a consequence, the procedures used in this research are believed to generate trustworthy and accurate findings.

## Limitations

Not applicable

## Ethics statements

The study involved human subjects. The students were distributed and informed about this study. The research consent form was signed by their parents and teachers.

## CRediT author statement

**Wan Athirah**: *Conceptualization and Writing*, **M. Fadzllah Zaini:** *Writing Original Draft Preparation*, **Mazura Muhammad:** *Protocol Strategy*, **Farahkhanna Rusli:** *Supervision,* **Haniff Md Tahir**: *Writing-Reviewing and Editing*, **Nurshafawati Ahmad Sani**: *Data curation*, **Habibah Ismail:** Text Collection and *Visualization,* **Suriati Zakaria, Md. Zahril Nizam Md. Yusoff, Norliza Jamaluddin, Dahlia Janan***: Instrument Expert.*

## Declaration of competing interest

The authors declare that they have no known competing financial interests or personal relationships that could have appeared to influence the work reported in this paper.

## Data Availability

No data was used for the research described in the article.

## Appendix Supplementary materials

Image, application 1

Image, application 2
