# Supplementary material for: Protocol for data collection on the language of poor students in Malaysia
Source: MethodsX. 2025 Sep 11;15:103616. doi: 10.1016/j.mex.2025.103616 (PMC12510012; doi:10.1016/j.mex.2025.103616)
Supplement: Supplementary file 1 [file mmc1.pdf]

KEMENTERIAN PENDIDIKAN MALAYSIA
BAHAGIAN PERANCANGAN DAN PENYELIDIKAN DASAR PENDIDIKAN
ARAS 1-4, BLOK E8
KOMPLEKS KERAJAAN PARCEL E
PUSAT PENTADBIRAN KERAJAAN PERSEKUTUAN
62604 PUTRAJAYA

TEL : 0388846591
FAKS : 0388846579

Ruj. Kami : KPM.600-3/2/3-eras(21986)
Tarikh : 21 Oktober 2024

WAN ATHIRAH ADILAH BINTI WAN HALIM
NO. KP : 961119085930

LOT 157295, RPT PEKAN LAMA, JALAN TANJUNG RAMBUTAN
31250 IPOH
PERAK

Tuan,

KELULUSAN BERSYARAT UNTUK MENJALANKAN KAJIAN :
PEMBANGUNAN INDEKS KEMISKINAN RELATIF MALAYSIA BERDASARKAN INTEGRASI SOSIOEKONOMI DAN KORPUS PERBENDAHARAAN KATA BAHASA MELAYU

Perkara di atas adalah dirujuk.

2. Sukacita dimaklumkan bahawa permohonan tuan untuk menjalankan kajian seperti di bawah telah diluluskan dengan syarat :

> **" KELULUSAN INI BERGANTUNG KEPADA PERTIMBANGAN PENGARAH BAHAGIAN PENDIDIKAN DAN LATIHAN TEKNIKAL VOKASIONAL, PENGARAH JPN DAN KEBENARAN PENTADBIR SEKOLAH/ KOLEJ VOKASIONAL TERLIBAT. "**

3. Kelulusan adalah berdasarkan kepada kertas cadangan penyelidikan dan instrumen kajian yang dikemukakan oleh tuan kepada bahagian ini. Walau bagaimanapun kelulusan ini bergantung kepada kebenaran Jabatan Pendidikan Negeri dan Pengetua / Guru Besar yang berkenaan.

4. Surat kelulusan ini sah digunakan bermula dari **21 Oktober 2024** hingga **21 Mac 2025**

5. Tuan dikehendaki menyerahkan senaskhah laporan akhir kajian dalam bentuk *hardcopy* bersama salinan *softcopy* berformat pdf dalam CD kepada Bahagian ini.Tuan juga diingatkan supaya mendapat kebenaran terlebih dahulu daripada Bahagian ini sekiranya sebahagian atau sepenuhnya dapatan kajian tersebut hendak diterbitkan di mana-mana forum, seminar atau diumumkan kepada media massa.

Sekian untuk makluman dan tindakan tuan selanjutnya. Terima kasih.

"BERKHIDMAT UNTUK NEGARA"

Saya yang menjalankan amanah,

Ketua Penolong Pengarah Kanan
Sektor Penyelidikan dan Penilaian Dasar
b.p. Pengarah
Bahagian Perancangan dan Penyelidikan Dasar Pendidikan
Kementerian Pendidikan Malaysia

salinan kepada:-

BAHAGIAN PENDIDIKAN DAN LATIHAN TEKNIKAL VOKASIONAL
JABATAN PENDIDIKAN JOHOR
JABATAN PENDIDIKAN KEDAH
JABATAN PENDIDIKAN KELANTAN
JABATAN PENDIDIKAN SARAWAK

* SURAT INI DIJANA OLEH KOMPUTER DAN TIADA TANDATANGAN DIPERLUKAN *
