# Supplementary material for: Protocol for data collection on the language of poor students in Malaysia
Source: MethodsX. 2025 Sep 11;15:103616. doi: 10.1016/j.mex.2025.103616 (PMC12510012; doi:10.1016/j.mex.2025.103616)
Supplement: Supplementary file 2 [file mmc2.pdf]

UNIVERSITI PENDIDIKAN SULTAN IDRIS
SULTAN IDRIS EDUCATION UNIVERSITY

BORANG : PENERANGAN DAN PERSETUJUAN RESPONDEN
FORM: RESPONDENT'S EXPLANATION AND CONSENT

Sila baca maklumat berikut dengan teliti. Sekiranya anda mempunyai sebarang pertanyaan, sila kemukakan kepada penyelidik.
Please read the following information carefully. If you have any questions, please ask the researcher.

TAJUK KAJIAN
RESEARCH TITLE

Pembangunan Indeks Kemiskinan Relatif Malaysia Berdasarkan Integrasi Sosio-Ekonomi dan Korpus Perbendaharaan Kata Bahasa Melayu
Development of Malaysia's Relative Poverty Index Based on Socio-Economic Integration and Malay Vocabulary Corpus

PENGENALAN
INTRODUCTION

Perbendaharaan kata merupakan sesuatu indikator penting untuk menilai semula indeks kemiskinan dalam kalangan masyarakat. Hubungan kemiskinan dan perbendaharaan kata ialah had kata yang berada dalam ruang mental setiap manusia boleh terbatas dan menyebabkan gangguan terhadap sosio-ekonomi masyarakat. Pemikiran masyarakat perlu diperkayakan dengan variasi kata yang boleh membuat mereka yakin menghadapi realiti dan mengubah kehidupan. Kajian ini berlandaskan tiga objektif iaitu (1) Meneroka data perbendaharaan kata bahasa Melayu golongan miskin berdasarkan pembinaan korpus pelajar; (2) Menilai impak kemiskinan berdasarkan kebersamaan sosioekonomi dan data perbendaharaan kata bahasa Melayu golongan miskin; dan (3) Membangunkan indeks kemiskinan relatif Malaysia berdasarkan integrasi sosio-ekonomi dan perbendaharaan kata golongan miskin. Kajian ini melibatkan 7 zon (Utara, Tengah, Selatan, Timur, Borneo, Pulau Pinang) yang melibatkan sekolah tertentu mengikut daerah. Penyelidikan ini menghasilkan Indeks Kemiskinan Relatif Malaysia berdasarkan integrasi Sosio-ekonomi dan Perbendaharaan Kata Bahasa Melayu. Di samping itu, hasil kajian ini juga membentuk satu Korpus Pelajar Perbendaharaan Kata Bahasa Melayu. Signifikan kajian ini memberikan nafas baharu terhadap penilaian indeks kemiskinan. Indikator perbendaharaan kata sangat penting dan menjadi garis dalam menentukan perubahan kehidupan golongan miskin. Jika anak-anak golongan miskin ini mampu menguasai perbendaharaan kata dengan baik, kemampuan komunikasi, interaksi, emosi malu, dan pendidikan dapat berkembang. Selari dengan polisi kerajaan untuk meningkatkan taraf sosio-ekonomi golongan miskin, bermula dari anak-anak ini kesan jangka masa panjang dapat dikurangkan dan kesan jangka masa pendek dengan mewujudkan pelbagai solusi atau kesedaran untuk mengubah gaya kehidupan dengan lebih baik. Secara tidak langsung menyokong usaha sifar kemiskinan menerusi SDG 1 (No Poverty).

Vocabulary is an important indicator to reassess the poverty index in society. The relationship between poverty and vocabulary is that the words in each person's mental space can be limited and cause disruption to the socio-economy of society. The thinking of society needs to be enriched with variations of words that can make them confident in facing reality and changing lives. This study is based on three objectives, namely (1) Exploring the Malay vocabulary data of the poor based on the construction of a student corpus; (2) Assessing the impact of poverty based on socioeconomic togetherness and Malay

vocabulary data of the poor; and (3) Developing a Malaysian relative poverty index based on the integration of socio-economics and vocabulary of the poor. This study involves 7 zones (North, Central, South, East, Borneo, Penang) involving specific schools by district. This research produces the Malaysian Relative Poverty Index based on the integration of Socio-economics and Malay Vocabulary. In addition, the results of this study also form a Malay Vocabulary Student Corpus. The significance of this study gives new life to the assessment of the poverty index. Vocabulary indicators are very important and are a line in determining changes in the lives of the poor. If the children of these poor people are able to master vocabulary well, their communication, interaction, emotional shyness, and education skills can develop. In line with government policies to improve the socio-economic status of the poor, starting from these children, the long-term effects can be reduced and the short-term effects can be reduced by creating various solutions or awareness to change their lifestyle for the better. Indirectly supporting the zero poverty effort through SDG 1 (No Poverty).

LANGKAH DAN PROSEDUR PESERTA
PARTICIPANT STEPS AND PROCEDURES

Tulisan
Writing

Kajian ini menyediakan instrumen untuk ujian bertulis yang telah dirangkakan dan dinilai oleh pensyarah yang pakar dalam bidang metodologi penyelidikan. Setiap peserta akan diberi tajuk karangan mengikut topik dan syarat patah perkataan. Setiap institusi diberi topik ‘cuti’ iaitu sekolah rendah dikehendaki menulis 80-120 patah perkataan. Bagi pelajar prauniversiti juga diberi topik ‘cuti’ dengan syarat panjang karangan sebanyak 580-650 patah perkataan. Seterusnya bagi pelajar kolej vokasional, semester 1 dan 2 panjang karangan hendaklah sebanyak 150-200 patah perkataan dan semester 3 sebanyak 350-500 patah perkataan. Setiap peserta Sekolah Rendah dan Kolej Vokasional semester 1 dan 2 diberi masa 1 jam. Manakala bagi peserta Kolej Vokasional semester 3 dan Prauniversiti diberi masa 1 jam 30 minit.

This study provides an instrument for a written test that has been designed and evaluated by a lecturer who is an expert in the field of research methodology. Each participant will be given an essay title according to the topic and word count requirements. Each institution is given a 'holiday' topic, which means that primary schools are required to write 80-120 words. Pre-university students are also given a 'holiday' topic with an essay length requirement of 580-650 words. Next, for vocational college students, semester 1 and 2, the essay length must be 150-200 words and semester 3, 350-500 words. Each Primary School and Vocational College participant in semester 1 and 2 is given 1 hour. While for Vocational College participants in semester 3 and Pre-university, 1 hour 30 minutes are given.

Lisan
Oral

Bagi instrumen ujian pertuturan bertopik, satu kotak undian disediakan yang diisi dengan pelbagai tajuk atau topik. Pelajar/murid akan mencabut undi, kemudian waktu diberikan mengikut tahap pelajar/murid tersebut untuk bercerita atau berkongsi idea berdasarkan topik yang diundi. Bagi murid sekolah rendah waktu diberikan selama 1-2 minit. Manakala, bagi pelajar prauniversiti dan kolej vokasional waktu diberikan selama 4-5 minit. Rakaman akan dilakukan bagi mengumpul data lisan.

For the topic speaking test instrument, a voting box is provided filled with various titles or topics. Students will cast their votes, then time is given according to the level of the student to tell a story or share ideas based on the voted topic. For primary school students, time is given for 1-2 minutes. Meanwhile, for pre-university and vocational college students, time is given for 4-5 minutes. Recording will be done to collect verbal data.

PENYERTAAN PESERTA
PARTICIPATION OF PARTICIPANTS

**Kriteria Pemilihan**<br>***Selection Criteria***	**Kriteria Pengecualian**<br>***Exclusion Criteria***
1. Kondisi organ pertuturan yang baik.<br>2. Pelajar yang sihat tubuh badan dan mental.<br>3. Murid tahun 4, 5 dan 6 atau tahap 2.<br>4. Murid B40 atau miskin tegar<br><br>*1. Good condition of speech organs.*<br>*2. Students who are physically and mentally healthy.*<br>*3. Students in years 4, 5 and 6 or level 2.*<br>*4. B40 students or extremely poor*	1. Pelajar yang mempunyai masalah pertuturan, iaitu gagap, dan kecacatan artikulasi.<br>2. Murid tahap 1 ke bawah<br>3. Murid M40 ke atas<br><br>*1. Students with speech problems, namely stuttering, and articulation defects.*<br>*2. Students level 1 and below*<br>*3. Students M40 and above*

MANFAAT YANG MUNGKIN
POSSIBLE BENEFITS

a) Murid / *Student*

Murid dapat meningkatkan kemahiran berbahasa dari segi pengolahan idea baru, penjanaan pengetahuan sedia ada dan motivasi.
Students can improve their language skills in terms of processing new ideas, generating existing knowledge and motivation.

b) Guru / *Teacher*

Mencipta pelbagai solusi dalam menambah baik struktur pendidikan bahasa, agar murid berpendapatan rendah ini dapat berfikir dan mengunjurkan bahasa dengan baik.
Creating various solutions to improve the structure of language education, so that low-income students can think and project language well.

c) Ibu bapa / *Parents*

Pelestarian kompentensi komunikasi yang berkesan melalui unjuran perbendaharaan kata yang baik dalam pertuturan seharian.
Maintaining effective communication competence through good vocabulary projection in everyday speech.

RISIKO
RISK

Peserta yang terlibat sebagai penyumbang data tidak mempunyai sebarang risiko dalam kajian ini.
Participants involved as data contributors do not have any risk in this study.

KERAHSIAAN
CONFIDENTIALITY
Ya / *Yes*

RUJUKAN
REFERENCE

Dr. Muhamad Fadzllah bin Hj Zaini
Ketua Penyelidik
FRGS/1/2022/SSI09/UPSI/03/1
Fakulti Bahasa dan Komunikasi
Universiti Pendidikan Sultan Idris
35900 Tanjong Malim, Perak.
Tel. 017-7559960
mfadzllah@fbk.upsi.edu.my

Ts. Dr. Suzita binti Ramli
Penyelaras,
Pusat Pengurusan Penyelidikan dan Inovasi (RMIC)
Universiti Pendidikan Sultan Idris
35900 Tanjong Malim, Perak.
Tel. 015-48797870
suzita@fsmt.upsi.edu.my

PERSETUJUAN / ***AGREEMENT***

Saya…………………………………………… No Kad Pengenalan. …………………………… beralamat……………………………………………………………………………………………………… ………………………..dengan ini bersetuju untuk mengambil bahagian secara sukarela dalam penyelidikan yang tersebut di atas *(kajian klinikal/percubaan ubat-ubatan/rakaman video/kumpulan sasaran/temuduga/ soal selidik).
*I…………………………………………… Identification Card No. …………………………… addressed to……… …………….. hereby agree to participate voluntarily in the research mentioned above *(clinical study/drug trial/video recording/target group/interview/questionnaire).*

Saya telah diberi penjelasan secara menyeluruh mengenai penyelidikan ini dari segi metodologi, risiko dan komplikasi (seperti tertulis pada Helaian Penerangan Responden). Saya memahami bahawa saya berhak menarik diri dari penyelidikan ini pada bila-bila masa tanpa memberi sebarang alasan. Saya juga memahami bahawa sebarang maklumat yang berkaitan identiti saya akan dirahsiakan.
I have been given a thorough explanation of this research in terms of methodology, risks and complications (as written on the Respondent Information Sheet). I understand that I have the right to withdraw from this research at any time without giving any reason. I also understand that any information related to my identity will be kept confidential.

Saya* berminat / tidak berminat untuk mengetahui keputusan kajian yang melibatkan saya.
I am interested / not interested in knowing the results of the research involving me.*

Saya setuju/tidak bersetuju untuk imej/gambar/rakaman video/ rakaman suara digunakan dalam apa jua bentuk penerbitan atau pembentangan. (sekiranya berkaitan).
I agree / do not agree to my image/photo/video recording/voice recording being used in any form of publication or presentation. (if relevant).

*potong yang tidak berkenaan *(*cross out what is not applicable)*

Tandatangan ……..…………………………	Tandatangan ……..………………………….
Signature (*Respondent*)	*Signature* (*Witness*)
Tarikh :…………………………………..…..	Nama :…………………………………..…..
Date	*Name*
	No. K/P: …………………………………..…..
	I/C No.

Saya mengesahkan bahawa saya telah menerangkan kepada responden ini sifat dan tujuan penyelidikan yang tersebut di atas.
I confirm that I have explained to this respondent the nature and purpose of the research mentioned above.

Tarikh ……..…………………………	Tandatangan ……..………………………….
Date	*Signature* (*Researcher*)
